# Anatase Titanium Dioxide Imparts Photoluminescent Properties to PA2200 Commercial 3D Printing Material to Generate Complex Optical Imaging Phantoms

**DOI:** 10.3390/ma14071813

**Published:** 2021-04-06

**Authors:** Tyler Dann, Jordan Raphel, Seth T. Gammon, Zachary Mastrovich, Tony Van Avermaete, Justin Jeffrey, Satish Adusumilli, W. Matthew Leevy

**Affiliations:** 1 Department of Biological Sciences, University of Notre Dame, 100 Galvin Life Science Center, Notre Dame, IN 46556, USA; tdann@nd.edu (T.D.); jraphel@nd.edu (J.R.); Zachary.Mastrovich.2@alumni.nd.edu (Z.M.); avanaver@nd.edu (T.V.A.); sadusumi@nd.edu (S.A.); 2 Department of Cancer Systems Imaging, The University of Texas MD Anderson Cancer Center, Houston, TX 77030, USA; stgammon@mdanderson.org; 3 Carbone Cancer Center, University of Wisconsin, Madison, WI 53705, USA; jjjeffery@wisc.edu; 4 Notre Dame Innovation Lab., University of Notre Dame, Notre Dame, IN 46617, USA; 5 Harper Cancer Research Institute, University of Notre Dame, 1234 N Notre Dame Avenue, South Bend, IN 46617, USA

**Keywords:** 3D printing, optical imaging, photoluminescence, imaging phantoms, optical properties, derenzo phantom, optical imaging

## Abstract

Selective laser sintering (SLS) is a prominent 3D printing modality that typically uses a polyamide (PA) powder as the substrate. One commercially available SLS material is known as PA2200, which is comprised of nylon 12 and titanium dioxide (TiO_2_) and is widely used to generate 3D-printed parts. Here, we report a unique optical photoluminescence (PL) characteristic of native, white PA2200, in which it yields a persistent, phosphorescence-type emission. An analysis of luminescence imaging data with emission measurements demonstrated that the anatase phase of the titanium dioxide additive is the source of the persistent PL properties. This characteristic of PA2200 enables advanced optical imaging applications, as demonstrated by luminescence imaging of an anatomical rat skeleton and a novel Derenzo-type phantom on a commercial image station. In summary, the light emission properties of PA2200 induced by the presence of anatase titanium dioxide open the door to a vast new array of complex optical applications, including the generation of imaging phantoms for training, calibration, and quality control.

## 1. Introduction

Three-dimensional (3D) printing, or additive manufacturing, is a powerful fabrication technique used to create complex architectures from a variety of materials. Unlike traditional methods of fabrication where material is removed (reductive manufacturing) or fit into a predefined space (molding), 3D printing is an additive process in which material is deposited with high spatial resolution in layers that are built up vertically. A myriad of 3D-printing techniques exists, which are generally classified by the way the material is delivered and bonded. Common examples include stereolithography (SL) and PolyJet printing, which use light to polymerize a liquid resin; fused deposition modeling (FDM), where molten filament is extruded and hardens in place via cooling; and sintering, where a laser selectively traces and fuses layers of powder resin (SLS) [1]. In particular, SLS has emerged as a robust modality due to part resolution, durability, and low cost. Polyamide 2200 (PA2200), a polyamide derived from nylon 12, serves as the most common substrate for this modality [2]. A distinct property of the native PA2200 material is the incorporation of titanium dioxide (TiO_2_) that imparts its white color.

Titanium dioxide is a white powder that has widespread use as a pigment in dyes due to its brightness, high refractive index, and resistance to discoloration [3]. In the commercial industry, it can be found in paints, printing inks, plastics, rubber, ceramics, cosmetics, and in electronic components [4]. TiO_2_ is incorporated into PA2200 to improve its flowability and absorption properties [5,6]. The global commercial production of titanium dioxide is millions of tons per year, with nearly 70% of commercial use as pigments [3]. As a pigment, it is known as titanium white, Pigment White 6, or CI 77891 [4]. Additionally, it is used in applications ranging from antimicrobial agents, photocatalysis, and energy storage [3]. In nature, titanium (IV) oxide (titanium dioxide, titania) can be found in two mineral polymorphs: anatase and rutile [5]. Both rutile and anatase phases have tetragonal crystal structures, with each a titanium atom coordinated with six oxygen atoms [7].

Optical imaging techniques that make use of light in the visible to near-infrared window have been used for a variety of research and clinical applications. In the pre-clinical research setting, bioluminescence (BLI) and fluorescence (FLI) imaging modalities have been developed to non-invasively monitor disease state progression and/or small molecule biodistribution in living mice [8,9]. In the clinical setting, new devices are under investigation for use in image-guided surgery and in the detection of diseased human tissues [10,11,12,13]. Additional technologies are being developed with potential for future clinical applications, including fluorescent peptides for in vivo detection of pathogens and for image-guided surgery [14], nanomaterials for in vivo transplanted cell tracking [15], and ex vivo biomarker detection systems [16]. In order to optimize image acquisition with these various techniques, it is critical to reproducibly calibrate each modality for parameters such as the linearity and homogeneity of excitation light in the field of view [17,18]. This can be achieved via the use of phantoms, which are tools used with imaging systems to reproducibly calibrate, correct, analyze performance, or train and educate personnel. While 3D printing has been utilized to create customized phantoms for imaging modalities such as X-ray Computed Tomography, magnetic resonance imaging (MRI), and positron emission tomography (PET) [19,20,21,22], its application to optical scenarios has been limited. Recent examples of 3D printed optical imaging phantoms include a mouse model [23], a mouse model with optical properties matching targeted tissues [24], a brain model with neovascular channels [25], and an anthropomorphic neonatal thoracic model with a pulmonary cavity [26].

Here, we present the unique photoluminescence (PL) properties of PA2200, which is comprised of polyamide-12 doped with titanium dioxide to yield a white SLS substrate. PL acquisitions were gathered to determine if PA2200 would photoluminesce with delayed emission of optical wavelength photons after “charging” with white light. The sustained emission of the material emulates a typical bioluminescence imaging (BLI) signal that is obtained by integrating several minutes of exposure on a charge-coupled device (CCD) chip, thus making PA2200 suitable for phantoms with this imaging modality. PA2200 displayed sufficient PL to detect with mainstream imaging equipment, and upon further analysis, it demonstrated very unique emission in the near infrared zone (≈800 nm). This PL signal was discovered to be long lasting, demonstrating ultra-long persistent luminescence on the order of 10s of minutes. To further showcase the breadth of capabilities of this material, a complex anatomical phantom was 3D printed out of PA2200 and photoluminescently imaged as a potential training and/or quality control aid. A Derenzo-type phantom, a common characterization and calibration phantom type in nuclear medicine imaging [27], was also prepared to measure the resolution of a given image station. Derenzo phantoms consist of triangular arrays of rods, aligned in a circular pattern, that are separated by a distance with the same order of magnitude as the rod diameter, with multiple rod diameters included in order to characterize the resolution of the imaging system [22]. Derenzo phantoms were originally designed using positron-emitting rods [27], but this phantom was designed using PA2200 as it was being used to measure luminescence resolution.

In summary, the optical properties of PA2200 described here, combined with its robust physical properties and mainstream use for 3D printing applications, make it a tremendously valuable material that can be utilized to create complex calibration and training phantoms for optical imaging devices in the lab and clinic.

## 2. Materials and Methods

### 2.1. 3D Printing with PA2200

Three-dimensional (3D) printed objects comprised of native PA2200 were obtained via a third party, Shapeways (Shapeways Inc., New York, NY, USA). Objects were exported as an “.stl” file, uploaded to Shapeways, and 3D printed using the SLS nylon (referred to as “versatile plastic”) in the polished white color. Shapeways, a private corporation, does not publicly share their printing parameters.

### 2.2. Preparation of 3D Files for Printing

To generate a bulk sample of PA2200 for testing, a square tube with 10 mm × 10 mm base and 40 mm length was created using the SolidWorks Professional software (SolidWorks Corp., Waltham, MA, USA) and printed as above. To showcase the training applications for PA2200 within optical imaging, a 3D-printed rat skeleton was produced as previously reported [28].

To demonstrate the ability of PA2200 as an optical imaging calibration phantom, a “Derenzo-style” phantom was created and manufactured. Using SolidWorks, a solid circular object was designed with six triangular subsections. Each subsection consisted of uniform, equidistant holes, ranging in diameter from 0.4 to 2.4 mm. The circular phantom was milled out of a flat sheet of black polypropylene (and constructed in a manner to allow for a disk printed out of solid PA2200 (60 mm diameter, 4 mm thickness) to be placed underneath, such that radiant photons would pass through the subsections of holes to be detected by CCD detector from above. 

### 2.3. Optical Imaging of PA2200 and Its Components

Samples of PA2200, anatase, and rutile titanium dioxide (Sigma Aldrich, St. Louis, MO, USA), and Nylon-12 (Sigma Aldrich) were charged for 10 s with ambient white light and placed in the light tight chamber of a Spectral advanced molecular imaging high throughput system (Spectral AMI HT, Spectral Instruments Imaging, Tucson, AZ, USA). Then, PL images were acquired with the following parameters: 30 s exposure time, 250 mm field of view, fstop of 1.2, focal-plane of 0 mm, open emission, and 4 × 4 binning. Then, the anatase titanium dioxide and the PA2200 were imaged under similar parameters with the exception of varied emission filters (530, 570, 610, 630, 670, 710, 750, 810, 850, and 870 nm) to create an emission spectrum. All data were plotted on GraphPad Prism version 7 (GraphPad Software, La Jolla, CA, USA).

### 2.4. Optical Imaging of Phantoms 

Both the training and calibration phantoms were imaged and tested for PL characteristics. The complex rat skeleton was charged with ambient light for 10 s and then imaged with the Spectral AMI HT with the following parameters: 30 s exposure time, 250 mm field of view, fstop of 1.2, a focal plane of 0 mm, open emission, and 4 × 4 binning.

The white PA2200 disk was charged for 10 s with ambient light, inserted into the Derenzo-type phantom, and imaged under the same parameters as the rat skeleton, apart from the binning. Four images were taken with varied binning parameters of none, 2 × 2, 4 × 4, and 8 × 8, while keeping all other parameters constant. All sets of images were analyzed for image resolution using the ImageJ software package, which was also used to make montages.

## 3. Results

### 3.1. PA2200 Component Analysis

**Figure 1:**
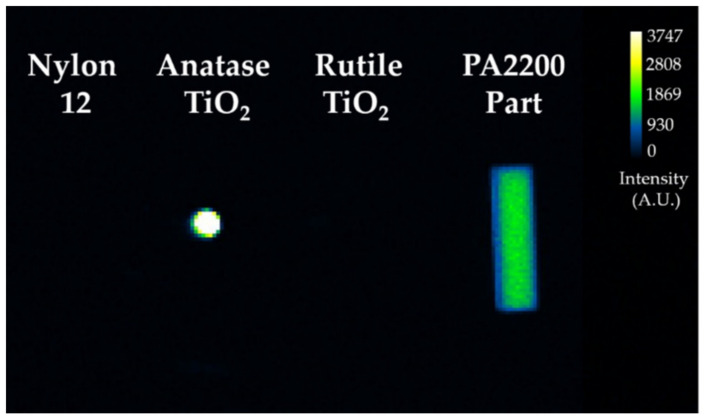
Photoluminescent signal of PA2200 and related components.

A subsequent optical analysis was undertaken of (1) nylon 12 polymer, (2) anatase TiO_2_ powder, (3) rutile TiO_2_ powder, and (4) a sample PA2200 part. Each material was imaged with the open filter in an AMI HT imaging station after being charged with ambient room light. Results showed that anatase TiO_2_ yielded significant PL, while the rutile form did not. Sintered PA2200 yielded about 50% of the PL intensity of anatase TiO_2_, while the native nylon 12 displayed no optical signal (Figure 1).

### 3.2. Photoluminescent Properties of PA2200

**Figure 2:**
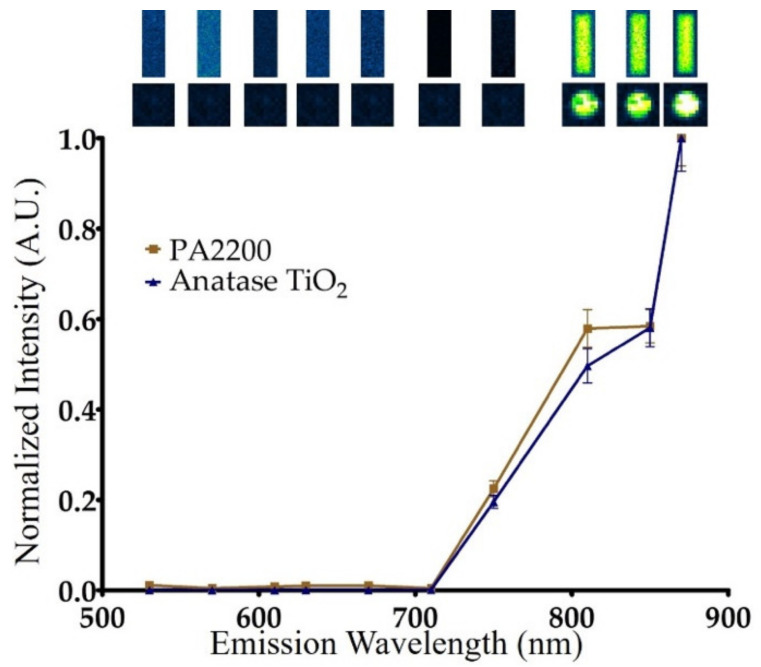
Normalized emission spectrum of the white PA2200 block and pure anatase TiO_2_ derived from photoluminescence (PL) images (shown at top).

The PA2200 SLS sample part exhibited sustained PL during optical imaging experiments on a commercial, CCD-based image station. A subsequent analysis was undertaken to determine the emission wavelength with strongest optical output. PA2200 was charged for 10 s with ambient room light and imaged in BLI mode with varying emission filters in front of the CCD to determine the wavelength with maximum optical output. The intensity values in each emission wavelength were plotted to yield a spectrum that revealed strong signal in the near infrared region (Figure 2). Anatase TiO_2_ powder was also imaged with the same parameters and showed an almost identical spectrum as noted with the PA2200. These results confirmed that anatase TiO_2_ is the photoactive species that gives PA2200 its optical properties.

### 3.3. 3D-Printed Optical Imaging Phantoms

**Figure 3:**
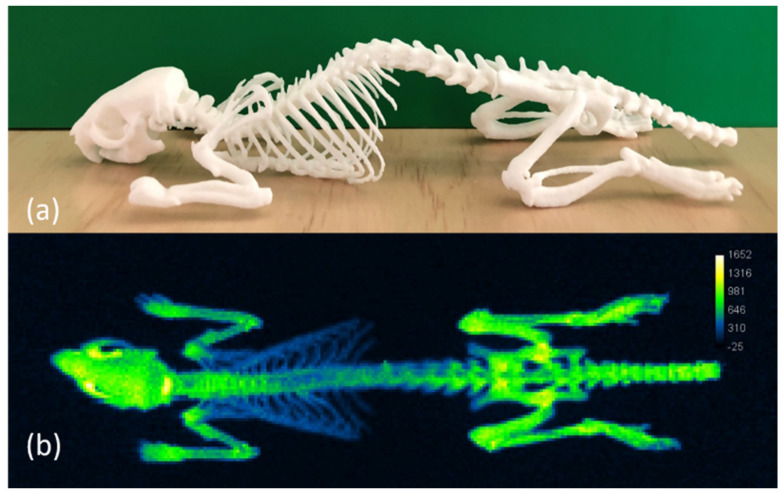
Three−dimensional (3D) printed PA2200 anatomical phantom of rat skeleton. (**a**) Photograph of a white PA2200 3D printed phantom. (**b**) PL image of phantom taken with open emission after preliminary charge with ambient light.

A rat skeleton was 3D printed in PA2200 as an optical phantom for potential use as a training aid and/or a quality control device. The modern SLS 3D printing technology deployed by third party Shapeways successfully created the complex object, which was robust enough to ship without damage (Figure 3a). This anatomical-style phantom, originally derived from X-ray CT scan data, displayed a strong PL signal under an open emission filter after being charged with ambient light (Figure 3b).

**Figure 4:**
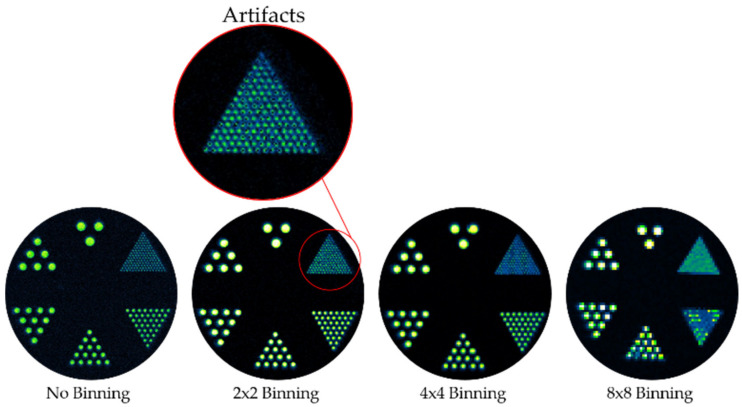
Optical imaging of Derenzo-style optical imaging phantom with varied CCD bin states. In the 2 × 2 binning setting, dead pixel artifacts were present, as highlighted in red.

To display the potential use of PA2200 as a calibration tool, a Derenzo-style phantom was designed to test the resolution capabilities of PL image collecting instrumentation. A disk with 5 mm thickness and 60 mm diameter was machined with six sets of hole patterns ranging from 0.4 mm (with 0.4 mm spacing) to 2.4 mm (hole and spacing). A disk comprised of white PA2200 was positioned underneath the hole grid such that light would only pass through the holes to be detected by the CCD detector. Optical images were collected at different bin states to demonstrate the relationship between binning and resolution (Figure 4). The phantom, when combined with the PA2200 disk, was able to show the resolution of pixels within different binning settings when photoluminescently imaged. In the No-Binning and 2 × 2 bin states, the 0.4 mm holes could be resolved, while bin 4 × 4 resolved the 0.8 mm holes, and bin 8 × 8 resolved the 1.2 mm ones. The utility of this phantom was further reinforced by the detection of an artifact in the 2 × 2 binning setting of the AMI HT; within this setting, the Derenzo images showed that there were dead pixels, as highlighted in Figure 4. The detection of dead pixels is important for imaging system characterization and calibration, as dead pixels can create additional experimental error by increasing the standard deviation of a region of interest measurement, thus increasing the sample size needed to obtain statistically significant results.

## 4. Discussion

Published reports indicated that PA2200 is a mixture of Nylon-12 and titanium dioxide, and the material derives its white color from the latter [5]. Given that TiO_2_ is commonly produced in two forms, anatase and rutile, the source of the optical behavior found in PA2200 was studied via component analysis. Imaging of PA2200 components confirmed that its long-lived PL characteristics were due to the anatase derivative of TiO_2_. While multiple crystal forms of TiO_2_ display photoluminescent properties spanning the blue to the NIR, only bulk anatase exhibits long-lived multiexponential decay of luminescence consistent with the slow migration of electrons through the bulk anatase TiO_2_ to electron traps [29,30,31,32]. When electrons combine with these traps (including interstitial Ti^3+^ and oxygen vacancies), NIR light is emitted [33]. Additionally, consistent with this model, the rutile form displayed an order of magnitude less (within machine noise) emission than anatase when charged with ambient light, as the electron transport and decay would have occurred with single exponential decay constants on the order of nanoseconds, which is much faster than the time required to place the phantom in the system and acquire an image. Emission spectra of both anatase TiO_2_ and PA2200 further supported that anatase TiO_2_ is the component that imparts PL properties to PA2200. Although both anatase and rutile TiO_2_ have tetragonal crystal structures and both can display photoluminescence, the mechanisms differ for the two polymorphs. Pallotti et al. provide detailed analysis of the differing photoluminescence mechanisms of anatase and rutile [34], which is beyond the scope of the research presented here.

It is uncommon to find 3D printable materials with photoluminescent properties that simulate an imaging modality. The discovery of near infrared PL properties of PA2200 was serendipitous, as these enable a wide array of phantoms for training and calibration. Here, two different phantoms were created as examples. First, since small animal optical imaging has robust usage in the pre-clinical research space, a rat skeleton was 3D printed with PA2200 and its luminescence captured on the Spectral AMI HT system. Since many training sessions on this type of machine would be conducted with live animals, this phantom provides a worthy substitute to reduce animal usage. A Derenzo-type phantom was also created and imaged for optical resolution of the AMI HT system. As noted in the Section 3, resolution decay at increased bin states was readily observable by use of the phantom. Such a QC process will be important as fluorescence-guided surgery and luminescent-guided radiation therapy gain broader adoption [35,36]. Unexpectedly, this phantom also uncovered a dead-pixel artifact in the bin 2 × 2 state, which motivated contact with the manufacturer to expedite a fix.

## 5. Conclusions

Three-dimensional (3D) printing is finding expanded use in creating affordable and facile prototypes and products in a range of fields, including medical imaging. Within luminescent imaging, optical properties can dictate appropriate material choices for a given application. Here, we present the photoluminescent properties of a common 3D printing material, PA2200, which is amendable to selective laser sintering. To demonstrate the use of these PL properties, a complex anatomical imaging phantom was printed with the white PA2200. This phantom demonstrated near infrared PL properties, enabling its use as a training or teaching device. The Derenzo-style phantom demonstrated the use of PA2200 for calibration purposes, even showing tangible results when uncovering an artifact within the AMI HT system. With the dual ability to create complex architectures that also display a PL signal in the near infrared zone, intricate phantoms can be custom made for individualized training that caters to the specific needs of the industry in which it is used. This opens the doors to creating unique phantoms for research and clinical use, allowing for the potential expansion of research within the PL imaging sector as a whole. Finally, the search for additional common 3D printing materials that may also have desirable optical properties that make them well suited as phantom materials for a range of PL imaging techniques can be opened up to the scientific community, enabling further possibility of research that intertwines the worlds of biological imaging modalities and 3D printing technology.

## Data Availability

The data presented in this study are available on request from the corresponding author.
